# The Role of Parent/Caregiver with Children Affected by Rare Diseases: Navigating between Love and Fear

**DOI:** 10.3390/ijerph18073724

**Published:** 2021-04-02

**Authors:** Beni Gómez-Zúñiga, Rafael Pulido, Modesta Pousada, Manuel Armayones

**Affiliations:** 1 Faculty of Psychology and Educational Sciences, Universitat Oberta de Catalunya, 08018 Barcelona, Spain; bgomezz@uoc.edu (B.G.-Z.); mpousada@uoc.edu (M.P.); 2 Education Department, Universidad de Almería, 04120 Almería, Spain; rpulido@ual.es; 3 eHealth Center, Universitat Oberta de Catalunya, 08018 Barcelona, Spain

**Keywords:** caregiver, parent, rare disease, chronic disease, qualitative research, role experience

## Abstract

In this paper, we propose a vision of the role of parent/caregiver with children affected by a rare disease. This vision is rooted in data obtained from our own research; however, our analysis and interpretation of this data have been subsequently checked against existing theoretical models. The research aims to explore how parents who look after children with a rare disease experience their role as caregivers and how they assimilate their role identity in this task. Semi-structured interviews were performed with parents of 10 children, and a qualitative data analysis was conducted using grounded theory. We have identified ten main categories using a grounded theory approach: stress, disorientation, insecurity, isolation, faith, trust, attention, communication with professionals, private proactivity and public proactivity. Our results also show that when parents perceive a greater burden due to looking after a child with a rare disease, the result is a change in the usual parental role. In our contribution, we offer a general outline of how parents build a role identity centred on caring for a child with a rare disease. We posit that this role identity is the outcome of the parents’ success or failure in gradually overcoming fear through love. We have conceptualized this process as navigating between love and fear.

## 1. Introduction

### 1.1. The “Role” and Its Relevance as a Construct in the Case of Parents/Caregivers

The sociological theory of roles states that anyone who takes part in a given social situation encounters specific expectations about how they should act [1]. The diversity of social situations is almost unlimited, but not all have the same weight in social life, nor are they all subject to the same control and predictability mechanisms. Situations such as teaching or medical care, and by extension, a multitude of professional practices—or priesthood and political representation—and by extension, different forms of leadership—place a heavy burden of expectations on the shoulders of those who take part in them [2]. For example, almost everyone expects women teachers, doctors, priests or mayors to behave in a certain way, have certain knowledge or show certain attitudes. Sometimes, what is expected of those who take part in certain social situations is set down in law beforehand, requiring them to embody specific positions in these situations, and anyone who does not conform to the role may be removed from the position held. In other words, there are roles subject to strong control mechanisms.

When analysing the roles that people hold, perform or exercise in society, there is one issue that takes on particular importance: the degree to which their acceptance of these roles is voluntarily chosen or freely decided. Becoming a parent is, more often than not, a conscious decision, something we deliberately seek. When we achieve parenthood, we naturally take on the parental role, that is, we accept the responsibilities that derive from it and we strive to meet the expectations that society and we ourselves place on us. Both—responsibilities and expectations—are included in our personal equation but the new needs that will arise and the new knowledge that we will have to acquire, and use are often left out.

For any given role, there are always a number of typical behaviours associated with it. They can be thought of as the hard core of the expectations associated with the role, in that they are behaviours that are presupposed or required of the role, and which may even be legally required of the person who takes on, embodies or performs the role [3]. Alongside these core behaviours, there may be other, less expected, not so “typical” roles that are subject to the variability of each personal case embodying the role [4]. This is what happens in the case of children with rare diseases. We define “rare diseases” as diseases that affect a small number of people compared to the general population and specific issues are raised in relation to their rarity. In the European context, a disease is classified as “rare” when it affects 1 person per 2000. A disease can be rare in one region, but common in another. To date, more than six thousand rare diseases have been identified and new ones are regularly described in the medical literature [5].

In the specific case of the role of a parent/caregiver of a child with a rare disease, for example, people are expected or required to show constant watchfulness of the child within the home. Furthermore, they would be forbidden to make any change to the prescribed medication guidelines, for example. A third type of behaviour would occupy a middle ground of permissiveness, such as proactivity in the independent search for information or active participation in associations, whose appropriateness or fit with the role will be judged in accordance with subjective criteria by the parents themselves or by the medical staff. Some doctors may “expect”—in the sense that it will not surprise them—parents to search the Internet or disagree with them about specific aspects, but that does not mean that they will believe that such behaviours are acceptable or in keeping with proper performance of their role.

There are different theoretical approaches to roles. To discuss the parent/caregiver role, we have chosen the symbolic interactionism approach, which views a subject’s role-derived behavioural patterns not as an automatic response to external pressures or stimuli, but rather as a subjective construct about themselves, about others and about the social demands that take place during their daily life [6]. The epistemological perspective of symbolic interactionism, in turn, is that which aligns best and most with the methodological approach we have followed, known as grounded theory. As researchers who have followed a phenomenological orientation, what interests us about the existence of a “role” is the way in which it is lived, perceived and experienced by those who assume or embody it. Symbolic interactionism provides the best conditions for understanding how and why learning the parent/caregiver role is full of ambiguities and conflicts, and how this learning takes place gradually through interaction with other human beings. It takes parents/caregivers some time to discover the expectations placed on them by their family, medical staff or society. However, once they have acquired this awareness, they learn not only to practise behaviours that are compliant with the role but also to rebel against certain expectations.

### 1.2. Theoretical Framework

Literature on parents with children affected by rare diseases is meagre compared to the literature on parents with a chronically ill child. However, a close inspection of literature about parents with rare diseases shows a degree of similarity in terms of parental experience high enough to permit solid comparisons. Our research is about a type of human experience, namely, being a parent with a child whose health state somehow makes parental care more intense. Once defined in those terms, “chronically ill” and “affected by a rare disease” are just two general types of situations in children lives where the parental experience under study exhibits its characteristics.

From this perspective, a first model that helps us analyse the parent/caregiver experience is that of Sullivan-Bolyai et al., who classify the responsibilities of the parent/caregiver of a chronically ill child in four categories:

(1) Managing the illness, which includes watchfulness, monitoring signs and symptoms, hands-on care, decision-making and problem-solving.
(2) Identifying, accessing, and coordinating resources, which means developing relations with institutions and medical personnel.
(3) Maintaining the family unit, which consists of integrating the management of the illness in the context of day-to-day family tasks.
(4) Maintaining oneself, which consists of caring for one’s physical, emotional, and spiritual health.

These four categories of responsibilities derive from the assumption of the parent/caregiver role and take the form of an extremely long list of tasks. When discussing the perception expressed by parents/caregivers about these tasks, the researchers suggest that these are “expectations”, that is, what is expected of them and could therefore be defined as the core of behaviours around which their role revolves [7,8].

A second model with which we can connect our vision of the role is that proposed by Pelentsov et al., focused on the needs of the parent/caregiver [9]. This is a complementary approach of enormous value for describing the role, insofar as it does not centre on the behaviours, expectations or knowledge associated with the role, but the needs that arise during the personal experience of the role. Thus, after performing a meta-analysis of 29 studies published between 1990 and 2014, Pelentsov et al. identified six types of needs in the parent/caregiver.

According to these authors [9], the needs most commonly cited were social needs (72% of papers), followed by informational needs (65% of papers) and emotional needs (62% of papers), with the most common parental needs overall being informed about their child’s disease, emotional stress, guilt and uncertainty about their child’s future health care needs, the parents’ own caring responsibilities, and the need for more general support. By identifying needs, it is possible to characterize a dimension of the role that is enormously important for a phenomenological perspective such as the one we are advocating, since it helps us to understand details of the daily human experience of those who embody the role. However, a framework such as that offered by Pelentsov et al. only shows a “snapshot” of limited value [9]. We should be able to say, at least, in which stages of the process (which begins even before diagnosing the disease) certain needs usually appear, which needs will remain constant, which will gradually disappear, which needs come and go, and what factors influence all of this. In other words, we need a theoretical framework that captures the role’s dynamics with regard to the needs that it generates.

To some extent, any need identified as such can also be described as a problem to be solved. In this respect, the proposal made by Van Scheppingen et al. is interesting [10]. These researchers built their vision of the role around the “problems” faced by parents/caregivers. Beyond the differences in these problems’ intensity or severity in individual cases, the authors pointed out that the core problems are always the same, and they broke them down into three themes, related to the child, the family, and the care providers. These themes comprised nine categories, including (1) the child being different, (2) the child suffering pain, (3) feelings of uncertainty, (4) restrictions on employment and leisure time, (5) difficulties in the organization of care, (6) never being off-duty, (7) family problems, (8) ignorance and lack of skills of care providers, and (9) resistance to difficult care.

The third model that we have used in our work was intended to capture the role’s processual dimension [11]. This is Raina et al.’s model of the caregiving process and the caregiver’s burden, which revolves around five constructs:

1. Background and context
2. Child characteristics
3. Caregiver strain
4. Intrapsychic factors
5. Coping/supportive factors
6. Health outcomes

Each of the first five constructs subsumes a series of factors that affect the sixth, caregiver well-being, although the authors accept that there may be additional factors not included in the model [11]. Its aim was to detect which factors had more weight as determinants of caregiver well-being, defined as the ability to cope with the burden (physical fatigue and stress) derived from caregiving. The idea was that knowledge of these factors would enable them to suggest effective supportive measures for caregivers. This model seems particularly useful, and we propose that the factors included in each construct can be reinterpreted as variables that affect how parents/caregivers self-perceive their role.

The first construct, background/context, is the framework in which the caregiving takes place and highlights factors such as the “family’s economic situation”, “the parent’s academic background”, “income level”. These are items that, for this model, influence stress levels. The second construct, child characteristics, refer to the specific illness suffered by the child, and the consequences derived from it in terms of impairment or disability. Citing a wealth of literature, Raina et al. suggest that:

… less severe disability will be associated with better psychological and physical health, lower ratings of caregiver demands, and more positive parental perception of formal services as being family-centered. Measures of child behavior problems assess behavioral issues such as conduct disorders, hyperactivity, emotional disorders, and somatization. These behaviors require surveillance, control and exertion on the part of the caregiver. We hypothesize that fewer behavior problems will be associated with higher caregiver self-perception, and better psychological health. [11] (p. 9)

The factors considered under the third construct, caregiver strain, include “caregiving demands” and “perception of formal caregiving”. According to the model’s authors, caregiving demands measure the daily demands on the caregiver, as well as conflict between the caregiving role and the primary caregiver’s occupational roles. In their view, fewer demands will be associated with higher scores on measures of self-perception such as mastery and self-esteem and increased levels of social support. For Raina et al., perception of formal care measures the extent to which caregivers report formal services as being family-centred, and higher scores on this construct will be associated with better psychological and physical health [11].

The model’s fourth construct, caregiver intrapsychic factors, is that which interests us most a priori, given our proposal’s phenomenological orientation. The factors included here refer to the caregiver’s internal state and, in describing them, the authors note that “for most individuals, caregiving constitutes a new social role; identification with the role often coincides with role incumbency and with the development of self-evaluation of how well one performs the role” [11] (p. 11). Self-perception is considered a vitally important intrapsychic factor and would be indicated by measures of self-esteem and a sense of mastery or ability to manage the caregiving situation. Referring to the abundant literature available, these authors hypothesized that higher levels of self-perception will be associated with higher perceived levels of social support, better family functioning and higher use of stress management strategies.

With the fifth construct, coping/supportive factors, Raina et al., refer to access to and use of supportive resources to cope with the caregiving situation [11]. This includes social support (friends, neighbours), the role of the extended family and stress management. Drawing from what many other studies have suggested, the model hypothesizes that higher scores in social support, family functioning, and stress management will be associated with better psychological health. In addition, higher scores in social support and family function will be associated with better physical health for the caregiver.

The sixth and final construct in Raina et al.’s model, caregiver health and well-being, refers to the “dependent macrovariable” within the model, that is, the caregiver’s health and well-being as a product of the influence that the factors grouped in the four previous constructs have on them [11].

### 1.3. Aim and Research Questions

This paper proposes a vision of the role of parents/caregivers with children who suffer from a rare disease. This vision is rooted in data obtained from our own research; however, our analysis and interpretation of this data have been subsequently checked against existing theoretical models, such as those mentioned above. Consequently, with our contribution, we aim to offer a general framework that allows a certain articulation between different theoretical proposals that already exist in the field of family/caregiver experience, viewed in the light of a body of empirical evidence obtained in the fieldwork.

With the goal of addressing the identity aspects of the role and the emotional experience of caring for children with rare diseases, we have posed the following research questions:

1. How do parent caregivers live their experience as caregivers?
2. What are the perceptions and emotions of a parent when they become a caregiver for their child with a rare disease?
3. What is the caregiver role identity of a parent of a child with a rare disease?
4. Does the parent of a child with a rare disease also perceive him/herself at the same time as a caregiver? Is there a conflict between the two roles?

## 2. Materials and Methods

### 2.1. Study Design

This article shows the results of qualitative data analysis as described in Gómez-Zúñiga et al. [12]. The corpus of text transcriptions of ten individual interviews has been analysed using ‘grounded theory’, employing open and axial text coding techniques. Initially, open coding of transcriptions was done by only one member of our research team, followed by a review of codes accomplished by the other three members. The grouping of codes into categories (axial coding) was jointly elaborated on by the whole team. The interviews were performed at the facilities of the FEDER (Spanish Rare Diseases Federation).

### 2.2. Participants

Interviews were semi-structured, with a script drafted beforehand by the researchers, and lasted between 60 and 90 min. Ten interviews were analysed (nine mothers and one father). The parents’ data collected from the in-depth interview were related to information about the children’s illness, followed by the problems they faced in caring for their children, both medically and emotionally. Some questions included: May I ask you first about your son/daughter? How is he/she feeling right now? How do you feel as parents? How do you feel in this active role? etc.

### 2.3. Ethical Issues

The first step in the interviews was to say how long the interview would last, followed by the signature of the informed consent, after which we asked the participants for permission to record the conversation. Participants gave their written informed consent to have a research team member interview them.

### 2.4. Grounded Theory

This article used the grounded theory as described in Gómez-Zúñiga et al., which is used in research on issues such as those concerning us [12]. The first step of the grounded theory analysis was to code the entire corpus. This initial coding was reviewed, and notes were formulated on the most significant codes. These notes or memos helped us group codes by their relationship, whether cause-and-effect, time-related, part of a whole, etc. Soon some codes began to emerge as more powerful, and others became weaker as we refined the categorization. Subsequently, those codes that included many others were renamed as categories.

The next step was to study each of the categories in greater depth to ensure that they were correctly constructed and to capture the relationships between them. This enabled us to find the core category that would provide the base for the other categories, and on which we could build the theory that would answer our research questions.

The core category reached its ‘data saturation’, as did the other categories when all their properties and dimensions were specified by the researchers. This gradual theorization enabled us to categorize and conceptualize the findings, find new data as concepts, as well as refining categories on the basis of their properties and dimensions.

## 3. Results

### 3.1. The Human Experience of Parent Caregivers (Research Question 1)

**Figure 1:**
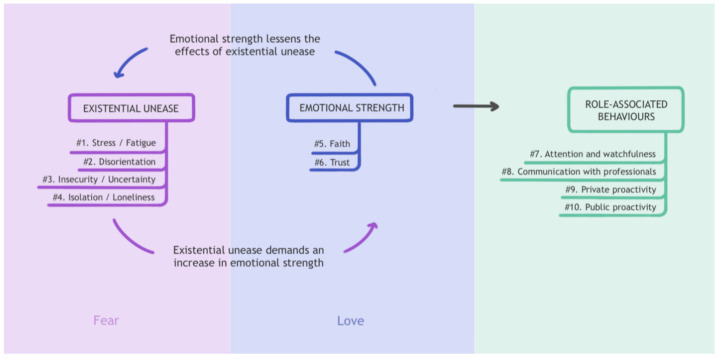
Flowchart of the parents’/caregivers’ role experience.

The analysis of the ten interviews we have conducted has enabled us to develop a map of the parents’/caregivers’ human experience (see Figure 1). We have drawn three zones on this map. Two of them, called “existential unease” and “emotional strength”—the lilac and blue areas of the chart, are the experiential reflection of the fear/love binomial and constitute, so to speak, the psychic substratum of the experience of the role, while the third corresponds to the visible behaviours linked to the role.

There is an important point to be made here. At any given moment along their experiential journey in the performance of the role, each parent/caregiver has their own particular combination of components of unease/strength. The fear/love makeup of this psychic substratum is constantly changing, subject to ups and downs, although it may show an overall predominant tendency or direction (greater inclination towards fear or towards love). However, there may not be any significant differences in the way in which role-related behaviours are performed from any particular tendency. In other words, an outside observer would be unlikely to be able to infer the nature of the role’s psychic substratum from the empirical evidence derived from the conducts observed. Watching and caring for their child, administering the prescribed medication, recording certain data, attending medical appointments or participating in association meetings, to name a few visible behaviours associated with the role, are things that are performed with a similar degree of competence both by parents/caregivers whose experience is mainly concentrated in the “fear” zone and by those who move within the “love” zone.

Existential unease—the “fear” zone—is always the starting point in the construction of the parent/caregiver role. The items that make up this zone may even appear before the child’s illness is diagnosed. The diagnosis usually intensifies them and, from then on, some of the items evolve in one direction and others in another, some more quickly and others less quickly, some almost disappearing while others remain relatively stable. This variability depends on the influence exerted by the items that make up emotional strength, the “love” zone, which can be posited as the antidote to existential unease. Here we find two items: trust and faith. When both are developed, the role’s experience is perceived with less suffering, as if the burden were less (even though the hours spent and the effort made have not changed). Trust and faith help give meaning to the role experience as a whole. The continual interaction between the two zones marks the direction that the role takes in each parent’s/caregiver’s experience. It may take different directions, as we will see further on, but first, we must illustrate the main items included in our chart’s three zones.

### 3.2. Existential Unease or the Substratum of the “Fear” Zone (Research Question 2)

The transcripts of our interviews were full of expressions related to what we have called “existential unease”. Here is a small sample of these expressions: “I understand that there are mothers who do not want to accept what their child has”, “I needed to disconnect from the disease”, the “horrible experience” of the first night, “the terrible experience of continuous epileptic seizures”, “you suffer an enormous personal strain”, “I’m exhausted”, “you don’t know what will happen in the future”, “you feel insecure when making decisions”, “you don’t want to accept what the doctor says”, “you feel as if you are alone to face everything that is coming at you”... One of the mothers who took part in our study said it with crystal clarity: “It’s hopeless, that’s the truth”. In the study performed by Eatough et al. [13], other parents said things like “this is a life sentence” or “your life is over”. This plethora of fragments and other highlighted comments in our interviews can be structured in four categories, although other categorizations are possible: 1. stress/fatigue, 2. disorientation, 3. insecurity/uncertainty, and 4. isolation/loneliness.

#### 3.2.1. Impacts on Daily Life

Existential unease is also caused by the impact that the situation generated by the disease has on different aspects of life. Here is a selection:

(a) Impact on family’s economic situation (see Domain 5 of Yuwen et al. [14], as well as the sub-category “maintaining family unit” in Sullivan-Bolyai et al.’s list of responsibilities and activities) [8]:
  *We had to decide that one of us had to stop working. Because looking after the child, especially considering how he was at that time... So what did we decide? “Who earns more? You, right? So I have to stop working”. So when I left, my employers arranged it so that I could receive unemployment benefit. I stopped working, and I devoted myself to looking after him. Because, apart from the first year, for example, I couldn’t go alone with him in the car, as he was constantly choking... I had to go with him in the back and my sister drove. Because we were also going to the hospital two or three times a week. Tests, appointments, whatever... and I had to revive him when he couldn’t breathe... and my sister was driving... for example, right? It was very complicated! So my husband had to work, and besides... he has always worked twelve hours, in fact, now, he also works twelve hours, you know? Because, of course, we had to have money to keep everything going...* [Interview 4]
(b) Impact on intrafamily relationships (see Domain 6 of Yuwen et al. [14]), especially when there are other children to care for, as already confirmed by the studies of Roper et al. [15]. In fact, their results already showed that when parents expressed higher levels of burden, their family relationships were rated less positively.
(c) Impact on the couple:
  *Well, there are two of us here. There are cases that...because I even know of one, that, that...in this case, the father... although sometimes it can also be the mother, I’m not saying that all fathers... that it’s always the fathers, but... the father said he couldn’t endure it any more with... the child... and he left. And he left her to go it alone, you know? I know that woman. And that woman said to him: “OK, leave then. You don’t want to have anything to do with your son, well, that’s okay, I’ll take care of him”. That didn’t happen to me... luckily! But it’s true that... when they gave us the diagnosis... the first thing I said to him was: “If you’re going to leave, do it now. I’ll take care of him”. Because... you see so many things, you say: “It could happen to you as well”. Well, for the moment, as I say, “he always goes to get cigarettes and he always comes back” [laughter].* [Interview 4]
(d) Impact on social relations:
  *You have no time for yourself... going out with friends becomes more complicated or impossible... It’s normal, people know that your time is devoted to your daughter and they don’t count you in for many activities, I guess they think it’s better for you, and they don’t call you like before.* [Interview 11]

#### 3.2.2. Explicit Fears

Fear is not only a diffuse substratum of the items that make up existential unease; it often manifests explicitly as it is, coming to the surface in different ways, such as fear of asking, of knowing, of making a decision, of the disease taking a turn for the worse, or of leaving the protected environment of the hospital. In our research, we have found various expressions of fear:

*It’s risky, we don’t dare, we don’t dare, we’re... we’re quite distraught. We dare not ask him, we are afraid to ask him. It’s very sad. […] Of course, the doctor doesn’t say to us that he doesn’t want us to ask questions. It’s our perception, it’s the feeling we get... I think our perception is of two people. Two people who, moreover, always think the opposite to each other. My husband and I... we always think the opposite, but we both have the same perception, we both have the same fear.*

*I want to know everything but I’ve also met other parents who have told me that they don’t ask the doctor anything. They avoid asking because they won’t be able to understand the answers properly but also because they are afraid of knowing too much. They prefer not to know [...] I know of several cases. So, of course, if they have as an example parents who don’t want to be given long explanations, because they suffer, because they are afraid…*

*And I can assure you that this doctor tried, he tried, but of course, we had already been with the other one for a long time, and I think it was a golden opportunity we lost... Because we were afraid to make the decision earlier. But, it’s too late now... to fix it…*

*Me, for example, I’m very worried about his liver, because of course, so much medication [...] I’m very afraid that it’ll affect his liver... And I don’t want that to happen! [Interview 9]*

### 3.3. Emotional Strength or the Substratum of the “Love” Zone

From what global psychological state or “psychic substratum” do parents/caregivers perform the behaviours linked to their role? In what proportion are the items of “fear” and “love” present? Existential unease will be fought, neutralized and relatively conquered if parents manage to acquire sufficient emotional strength. In any case, as we have said earlier, parents have no choice but to show certain conducts before they manage to increase their emotional strength. Existential unease demands that parents increase their “reserves” of emotional strength. If this demand is not met, the effectiveness of their caregiving may be undermined, and in the worst-case scenario, it would bring about role collapse, that is, the parents would give up, run away from the situation and/or suffer serious consequences for their psychosocial stability. In order to meet the demands that existential unease presents to them, parents develop two intimately related but analytically separable qualities: trust and faith.

Category 5: Faith

In the context of our area of study, faith can be understood as the intimate conviction, more intuitive than based on verifiable facts, that “things are going to be all right”, which translates into ideas such as “I am sure that I can handle all this”, “my child will not lack nothing”, “we are in the best hands”, “a treatment will be found for this illness”, and other similar ideas. In other words, faith goes beyond mere trust and opens a door of hope while providing the emotional energy needed to “keep going” and not fall apart.

“Faith” takes us into the spiritual dimension of the experience of the parent/caregiver role, insofar as it is intimately connected with the search for meaning. We can define this search as the effort to accept and understand the reality of the illness and the personal and family life situation caused by it. The nature of this search and of faith itself may be due to specific religious beliefs or to a spiritual attitude in life that is not linked to any religious creed. However, we could say that “faith” is not inherent to the parent/caregiver role—as opposed to trust, which is—although its presence no doubt enhances emotional strength.

In her study on the influence of spirituality and religious beliefs on the experience of parent caregivers, Speraw noted that most participants expressed surprise that a nurse (as the researcher herself was) should be interested in spirituality [16]:

No one who mentioned nurses indicated that these professionals had ever (a) discussed spirituality with the family, (b) recognized that children with disabilities might have a spiritual life of their own, (c) understood that spirituality or membership in a faith community would be important to the family, or (d) appreciated that the support of a faith community could help provide inner consolation as well as enhancement of coping. (pp. 226–227)

Just as there is a big difference between resignation and acceptance, there is also a big difference between acting out of ethical duty (inherent to the role of a parent) and acting out of unconditional love. This is something that goes beyond the role, as someone could care for a child with a rare disease moved by that unconditional love without being the parent. All this comes to mind in relation to a dimension of the parent/caregiver role that seems particularly important to us: the search for a meaning for life in the personal caregiving experience. This was clearly expressed by Noonan and Tennstedt [17]:

One resource that may help explain why some caregivers fare better than others is a construct known as meaning in caregiving, defined by Giuliano, Mitchell, Clark, Harlow, and Rosenbloom (1990) as “positive beliefs one holds about one’s self and one’s caregiving experience such that some benefits or gainful outcomes are construed from it”. (p. 785)

For these authors, the ability to frame caregiving as meaningful and positive will explain significant variation in well-being among caregivers even after contextual and stressor variables have been controlled. Thus, among the factors not included in Raina et al.’s model, one of them is meaning, which we could define as “the attribution of a deep meaning to what we do, to the point that this meaning gives order and coherence to our life” [18]. In our opinion, any theorization on the parent/caregiver role should emphasize this dimension of meaning, a term that…

… has been attached to a variety of issues involved in informal caregiving: the motivation for becoming or remaining a caregiver; how one describes one’s caregiving; the impacts of caregiving; the appraisal of how burdensome it is; the efforts to make sense out of one’s experience; the meaning-making categories utilized; and specific positive aspects that may emerge from these attempts. (p. 44)

Durá-Vilá et al. aimed to show how parents’ unexpected experience of an unusual offspring is interpreted as a great gain, indeed one which is imbued with sacred meaning that requires explanation in religious or cosmic terms [19]. They showed how these beliefs strongly influence the parental care for the child and the implications for the quality of relationships and the sacred meaning of the child for the family life-cycle. This study illustrates how much of the parent’s understanding of their child’s intellectual disability, as well as their attitude and way of coping, are influenced and based on their religious/spiritual beliefs.

However, Durá-Vilá et al. indicated that these parents’ religious/spiritual beliefs can be viewed as having both positive and negative consequences for their own psychological well-being as well as for their children [19]. On the positive side, the beliefs appear to contribute to an increase in the families’ cohesion and resilience. Their faith provides an interpretative framework for understanding, accepting and viewing their child’s disability in a more positive light. On a more negative side, there is a need to consider the impact of the relationship between the parents and children on the children’s psychosocial development. The high emotional over-involvement is recognized by characteristics such as intense affective expression in the interview, over-protectiveness and self-sacrificing behaviour.

Chivukula et al. point out that spirituality was a significant predictor of emotional, physical, developmental, and social burden. According to these authors, the relationships dimension of spirituality predicted four dimensions of caregiver burden, indicating that maintaining and valuing relationships had an effect on their caregiver stress, probably because relationships provide emotional support and tangible support at the time of crisis. Thus, including spirituality in coping styles can decrease caregivers’ perceived burden and improve their well-being [20].

Although we have no empirical data of our own in this regard, we believe that for the role experience to be stable within what we have termed the “love zone”, where trust and faith provide deep meaning to the everyday reality that they are living, parents/caregivers would find great help in compassion- and mindfulness-based interventions (CMBIs) and therapies such as those discussed by Cousineau et al. [21]. According to these authors, these therapies…

… hold promise to support parental resilience by enabling adaptive stress appraisal and coping, mindful parenting, and perhaps crucially, self-compassion. These contemplative modalities have recently been expanded to parents of children with chronic illness, building on successful applications for adults facing stress, chronic pain, or mental illness, and for healthcare professionals in response to caregiver burnout resulting from their work. (p. 1)

Category 6: Trust

Trust is a type of relationship that the parent/caregiver establishes with certain agents or objects, by virtue of which he/she feels more confident in the overall handling of the situation. Trusting the medical staff, the hospital as an institution or a particular treatment or technique reduces the anxiety generated by uncertainty, but only to the extent that the parent/caregiver has evidence on which to base their trust. We could say that trust is a faith supported by evidence, whereas faith is a relatively “blind” trust.

As emotional strength grows, existential unease becomes an attitude of vital engagement in the face of an unexpected difficulty that must be integrated into the daily routine. Depending on the case, this attitude’s general tone may take the form of resignation, acceptance or personal challenge, among others. If there is no increase in emotional strength, existential unease gives way to what one interviewee described with the words “it’s hopeless”, a situation in which the initial existential unease becomes “getting used to suffering”.

In any case, no matter how much emotional strength the parents may have accumulated, they must remain ever vigilant, as the items from which the unease feeds are always latent, even if their effects have been attenuated. Various circumstances can cause them to re-emerge through the emotional cracks that are constantly opening in the daily management of the disease. However, it is very unlikely that all these qualities of the “fear” zone (#1 to #4) will reappear together, or with the same strength as at the beginning. Consequently, there is no possibility of returning to the stage of existential unease when this unease was at its height. In fact, this possibility can be ruled out altogether for those parents who have already developed their “role” as caregivers.

### 3.4. Role-Related Behaviours (Research Question 3)

Faced with the reality of their children’s disease, parents are forced to take action immediately, before they can build up enough emotional strength. Right from the very beginning, when the existential unease has already begun, they start to show behaviours related to the parent/caregiver role. The disease cannot wait, and parents must care for their child, regardless of the inner suffering they are experiencing and regardless of whatever combination of “fear” zone items predominates in their experience.

Parents/caregivers materialize their role in specific conducts that we have classified in four categories within our theoretical framework: cat. #7. attention and watchfulness; cat. #8. communication with professionals; cat. #9. private proactivity, and cat. #10. public proactivity. The first two are inherent to the role and they are constantly deployed, which means that parents must perform them always. The last two can occur with varying degrees of intensity or engagement on the part of the parents, which means that they are dependent on their personal choices. In other words, attention/watchfulness and communication with professionals are behaviours that parents must develop, no choice about it, from the very beginning and will never abandon. On the other hand, being more proactive or involved in associations and other public activities related to the disease is optional or intermittent, depending on various factors.

Ultimately, a role is a set of behavioural expectations that some people place on others or on themselves according to the position held within a given situation. In this respect, what is expected of a parent/caregiver? Who expects it? Do they expect it of themselves? Do they impose it on themselves as an obligation?

#### 3.4.1. Attention and Watchfulness (Cat. #7)

Watching the child night and day, observing their gestures, their behaviour or recording information about their evolution, all of this makes up the central core of the behaviours associated with the parent/caregiver role:

*Our son usually has perfect blood tests, I’m telling you. No, it’s true, everything is normal. Why? Because you’re keeping a constant watch. You look at the blood tests and you say, “Oh, he’s low in this!” “Let’s give him something”, or we talk to the doctor, “What can we give him?”, if it’s not something you can buy. “What can you...” “Ah, well, this would bring the level back up”. In other words, all this work so he has a blood test that’s within normal levels, even if he has the disease, because you’re giving him quality of life.* [Interview 6]

Attention and watchfulness take the form of a myriad of responsibilities and activities such as those outlined by Sullivan-Bolyai et al. in their comprehensive list of daily tasks associated with the parent/caregiver role for chronically ill children [8]. These authors were able to specify 49 tasks/responsibilities within the category “Developing a working knowledge of the treatment plan”, grouped into eight sub-categories: monitoring condition and behaviour; interpreting; providing hands-on care; making decisions; developing care routines; making treatment adjustments/problem-solving; guiding or coaching children in self-care: striving for interdependence versus control; and teaching others how to care for the child (e.g., teachers, sitters, family members).

#### 3.4.2. Communication with Professionals (Cat. #8)

The exchange of information and opinions is a crucial aspect in a positive communicative relationship between parents and doctors, insofar as it fosters a sense of mutual trust. As one of our interviewees said in a previous publication [12]: “I need to know everything and more, much more, and, and, and... and I love it when a doctor explains things to me and explains them well. I love it! When a doctor explains something well, when you understand and enjoy it, even if it’s something negative about your kid, well, it gives you a clearer idea of what’s happening to your child.”

Many of these behaviours correspond to the 20 responsibilities and activities that Sullivan-Bolyai et al. distribute within the sub-categories “negotiating health-related issues”, “negotiating an emergency plan”, and “providing coordination of care” [8].

Collaborative behaviour with the doctors is inherent to the parent/caregiver role, in the sense that all the people involved expect such behaviour. However, it is obvious that there may be disagreements as to the limits of such collaboration, or simply what is and what is not collaboration.

*Umm, I don’t want to give the doctor more work than he already has. If I can go, if I have to find out myself how to go somewhere to have it printed or go to a website to download it, I don’t mind, I do it, because I don’t want to give the doctor more work than he already has. They already have a lot of work. I want to make their life easier…*

*I think they do try* [collaborative work between doctors and parents], *but sometimes they just can’t find the time. The way the public health system is here, I think that, many times it is not so much not wanting to as not having enough resources.*

[T]*he intention is to collaborate as much as possible, to put ourselves at the service of whatever the doctors may need, that is, always, not try to jump ahead and do things that... that... maybe might bother the doctor, but otherwise... Above all, to convey our total wish to help and collaborate... that’s how I see it.* [Interview 10]

The caregiver’s role in decision-making can be controversial. Parents and doctors may have different opinions about how far each party’s role goes and the advisability or not of sharing certain decisions [12]:

*I know there are families who prefer to just follow the instructions given by the medical team. And that the doctors guide them totally on what to do... I don’t feel comfortable with that. I feel comfortable making my own decisions, because whether it goes right or wrong... if I have to... if I have to think that I knew that that was not what I should do but I did it because they convinced me...* [Interview 7]

#### 3.4.3. Private Proactivity (Cat. #9)

We can define proactivity as a general attitude shown by parents towards their child’s disease. This attitude consists of an ongoing threefold desire to know more about the disease, to improve their proficiency in managing the disease as effectively as possible, and to pool the effort and experience of all those affected by the disease, whether they be patients, family members, medical professionals or researchers. The behaviours derived from these desires can develop within each parent’s personal or private space, or they can develop in a shared public space, for example, in associations; hence our distinction between private and public proactivity. Many of these behaviours would fall under the category “Identifying, accessing, and coordinating resources” in Sullivan-Bolyai et al.’s list [8]. This category includes behaviours such as “takes initiative in seeking resources”, “seeks resources wisely: casts a wide net”, “seeks authoritative advice when appropriate”, or “weeds out erroneous, inaccurate or inadequate advice (including internet)”. Information-seeking is perhaps the most typical private proactive behaviour:

*We are always looking for information, always, always, always. And we try to look for it on websites, such as Orphanet, Wikipedia, well, Wikipedia not so much, you can’t rely on everything that’s written there... Websites that we believe to be safe and reliable. So what happens? We try to assimilate all this information as best we can, because we’re not doctors, but... this information makes us ask ourselves questions, and then we could take these questions to the doctor to clear up doubts, but we cannot... we cannot... if we ask the doctor questions, the doctor feels attacked, that’s our feeling.* [Interview 2]

The parents’ expertise, gained from their incessant search for information, sometimes leads them to put themselves on the same level as the professionals. As one mother told us:

*It’s funny, once it happened to me that they were going to give* [my son] *general anaesthetic, I don’t know if it was at Hospital X or I don’t know where, and I told the anaesthetist: “Look, he’s got this, he’s got that,” and he said to me: “Are you a doctor?” “No, I’m a mother, you know?”* [Interview 5]

#### 3.4.4. Public Proactivity (Cat. #10)

Proactive parents/caregivers often include active participation in associations formed around their children’s illness or rare diseases in general in their repertoire of role-related behaviours. For some parents, this public proactivity is the best option:

*In the end, I think that if we all open our eyes a little and look at other cases and learn from other families and learn from other associations how they work, we can move forward and learn more. We can’t just focus solely on my son, my son, my son’s illness...* [Interview 6]

This is particularly the case when they reach a saturation point with the information obtained from private searches:

*There are times when so much information, I don’t know if it’s good, you know? Maybe it’s better to filter it through associations or... Of course, ... yes, I’ve seen it in parents, but of course, I mean, if you want to know everything, ... I mean, it would be a daily job, you know? But it’s not just here, you start looking at... You start looking at the American Association, the English Association, see what they’ve done there…*

*But everything we see is very hard and difficult. So we said: “Well, let’s form a movement, create an association, because in Spain there is nothing and we can start getting things done”.* [Interview 6]

Public proactivity, in the form of involvement in associations, can not only complement or provide alternatives to private proactivity, but also a source of conflict with doctors about whether it is appropriate behaviour for the role from the point of view of some professionals:

*So what you usually do is either look for information on the internet or look for an association, if you are lucky enough to find an association close to you... to where you live. And, strangely enough, it is in the associations where you can get more information, more treatments, and not from the doctors themselves. From what we have seen, doctors are reluctant to tell us certain things. I don’t know if it’s because sometimes things aren’t approached the right way, or if it’s another reason, but there are some doctors who don’t have a good opinion of the associations or what they’re supposed to do.* [Interview 8]

### 3.5. One Role and Three Trajectories (Research Question 4)

If we remember the models that we used at the beginning—with several more having similar features have been published in the last 20 years—there are many and very diverse factors that influence the process of integrating the parental/caregiver role. Some factors influence continuously, and others influence intermittently or at certain times or stages. All of them describe the role’s process, but the only thing that explains that process, that is, the only thing that explains why some parents take one direction or another is that each parent has a personal, unique way of perceiving, analysing and interpreting these factors’ influence and managing the interaction within the privacy of their experience. So, the key is to be found in what Raina et al. called “intrapsychic factors”, and this is the theoretical model that we use as the basis for our analysis of the parental and caregiver roles [11].

**Figure 2:**
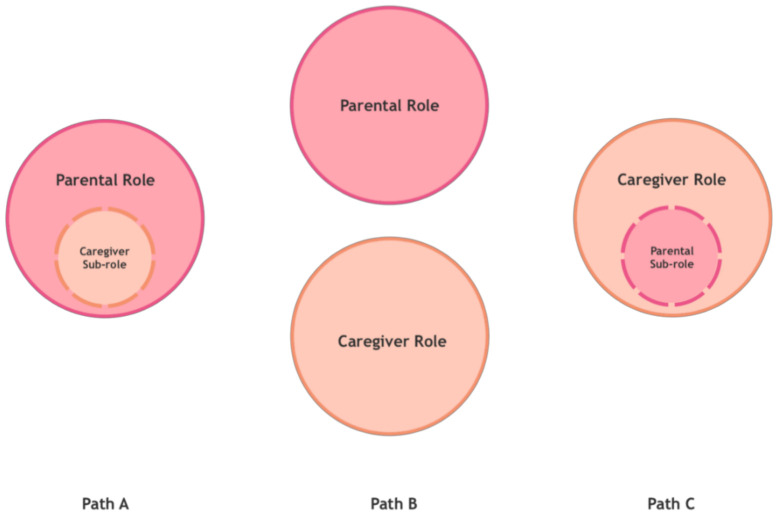
Changes in the parental role.

When looking after a child requires such dedication from the parents (time, physical and emotional energy, attention, etc.) that it comes to be perceived as a much greater burden than what we typically associate with parenting, it causes changes in the parental role. These changes can take three possible paths (see Figure 2), which in turn can be followed with relative success or failure, measured in terms of the parents’/caregivers’ perceived satisfaction or well-being.

The first path consists of an (A) extension of the parental role, so that being a caregiver to the child—parents would tell us—is simply something that, although we would prefer it not to have happened, is part of our parental role. This path will be more or less successful to the extent that parents accept and integrate the demands of the (sub)role of caregivers in their parenting. Basically, it does not interfere with their view of parenting or how they experience it.

The second path consists of dissociation of roles, so that, these parents would tell us: “I perceive and practice a (B) separation between the parental role and the caregiver role”, with different degrees of well-being or discomfort as a consequence of this separation.

However, there would be a third possibility, because the course of both paths can lead to an (C) oversizing of the caregiver role. This can happen when what was initially an extension of the parental role has grown to take over certain areas of parenting and the parents’ own personal development: I become a father/mother who barely enjoys parenthood beyond the special care of my child. This can also happen when what was initially a separation of roles has become a situation in which one of them, the parental role, becomes a sub-role: I become a caregiver who is only a parent part-time. Currie and Szabo describe certain cases of parents who sacrifice themselves to the caregiving role and become therapists and caregivers to their medically fragile children at the cost of losing themselves as parents [22]. Skaff and Pearlin had already described similar processes in which the caregiving role “engulfs” the person who embodies it, leading to a loss of the sense of self [23].

When the external expectations about showing behaviours associated with the parent/caregiver role match the expectations of the person who embodies it, the role develops appropriately and is accepted by all. However, when this does not happen, a role conflict arises. This is more likely to happen in paths A and C above. When external and personal expectations about the behavioural patterns associated with the role are not defined, we talk about role ambiguity. Both role conflict and role ambiguity can cause what Backman and Secord define as role strain [24]. When applied to our field of study, this concept would be defined as the discomfort experienced by parents when trying to meet the behavioural expectations assigned to their role as caregivers.

Being a parent, or being a professional caregiver, is much easier than being a parent and caregiver at the same time. Not because the mere sum of two roles arithmetically increases the burden they impose, but because one of the roles—the parent—tends to extend beyond what is considered “normal”, to the point that it becomes merged with the other role—the caregiver; this merger can cause turbulences (not to be confused with existential unease, which is the baseline psychological state in the process’s early stages). In some cases, the merger causes the caregiver role to literally “engulf” the parent role, and this can lead to identity crises and intrapersonal and intrafamilial conflicts. In such cases, a negative sense of self-worth in the caregiver (sub)role leads parents to perceive themselves as failing “by extension” as a father or mother, which is very unfair and very painful.

## 4. Discussion

In this paper, our main goal was to describe the role identity aspects and the emotional experience of caregiving. We have delved into how caregiving parents live their experience as caregivers, what their perceptions and emotions are, what their role identity is, and how the role of parent coexists with that of a caregiver.

Working from the results obtained, we have built an overall framework that explains the parents’/caregivers’ human experience. We have proposed “existential unease” and “emotional strength” as experiences that arise from fear and/or love and which shape the role experience. We have also described some findings on role-related behaviours.

Based on the interviews we have analysed, our contributions to “existential unease” illustrate many of the items cited in the literature, for example, those compiled in Thorton and Travis’s Modified Caregiver Strain Index [25]. However, our list of items that make up existential unease (#1, #2, #3 and #4) matches the comprehensive list of “common problems” experienced by caregivers provided by the American Psychological Association: depression, anxiety, subclinical stress, high rates of negative affects including guilt, sadness, dread, worry, ambivalence about care, witnessing the suffering of relatives, becoming easily irritated or upset, feeling isolated or abandoned by others, anticipatory grief, fatigue, sleep problems, risk of illness and/or injury, weakened immune system, lower self-rated physical health compared to that of others of the same age and sex, adverse changes in health status, greater health services use and medication use, dysregulation of stress hormones, financial strains, relationship stress, loss of time for self-care (e.g., sleep, diet, exercise), and reduced quality of life.

Based on the categories or domains we have included within “existential unease” (stress/tiredness, disorientation, insecurity/uncertainty and isolation/loneliness), we can highlight some recent studies that have focused on these items separately, such as Craig et al.’s studies on stress/tiredness [26], or Lundberg et al.’s study on insecurity/uncertainty [27]:

The period before the diagnosis was filled with uncertainty. How Do We Cope (Knowing How)? Parents’ comments about the initial period were also characterized by a need for knowing how to deal with this new situation. (p. 523)

There is also Currie and Szabo’s study on loneliness/isolation [28]:

“Very few people get what our life is like”. Parents expressed misunderstanding and lack of comprehension from family and friends about the reality of caring for their children. Instead of overcoming adversities and displaying resiliency as within normative narratives, parents revealed the struggle with coping with day-to-day experiences. “It’s very isolating”. (p. 6)

In line with our results, we found Yuwen et al.’s study on the experience of parents/caregivers with children affected by juvenile idiopathic arthritis (JIA) to be particularly interesting [14]. They followed our methodology (grounded theory), with a sample very similar to ours (eight mothers and one father in their case, for nine mothers and one father in ours). After analysing the content of these nine in-depth interviews, they described the whole spectrum of items of what we call “existential unease”, and which they called “struggling in the dark”. The core category “struggling in the dark” receives input from six domains in these authors’ study. The description they give of these six domains is very similar to the four domains we have included in “existential unease”:

- Domain 1. Not knowing (#2. Disorientation and #3. Uncertainty in our framework). Parents felt blindsided by the huge amount of information they received about the diagnosis and the treatment options. They were expected to make treatment decisions for their child despite their difficulty processing the information and feeling scared and unsettled about the unpredictability of the disease.
- Domain 2: Reaching out in the dark (#4. Loneliness in our framework). Parents felt that nobody could understand them and felt totally “alone and in the dark” when their child was diagnosed.
- Domain 3: Working out the kinks to stay on top to manage (this connects with #1. Stress and #3. Insecurity in our framework). Parents described the experience of seeing their child “getting sick all the time” from JIA-related symptoms or medication side effects. Parents felt that the medication that was supposed to treat JIA was making their child sicker.
- Domain 4: Feeling my child’s pain (#1. Stress in our framework, in this case, due to empathetic suffering). Children frequently had blood draws, received injections into their joints and experienced pharmacological side effects. Parents felt hurt when their child was hurting, and some even felt they experienced the pain themselves.
- Domain 5: Feeling drained by the whole process (#1. Stress/Fatigue in our framework). Challenging situations continued to happen one after another after the JIA diagnosis. Parents described JIA as “consuming [their] life”, and felt emotionally, physically, and financially drained. Parents had to take time off work to care for the child, and at the same time had to work extra hours that cut into their leisure time.
- Domain 6: Being hard on the entire family (this connects with what we have said about “impacts”, in Section 3.2.1.). JIA did not only affect the child, but also impacted the parents, siblings, and the relationships between family members. Parents felt that every aspect of their lives was affected by JIA and they had to change daily routines to deal with it.

In the psychic substratum that we described in the “Results” section, consisting of a combination of unease/strength-related items, the parents’/caregivers’ experience will evolve in accordance with the trend followed by the fear/love dynamic. Fear underlies all the categories of existential unease, and to combat it, parents develop both trust (#6) and faith (#5), which lend emotional strength to their experience.

Previously, we had described trust as a core concept in parent/professional relationships, because we understood that successful or fluid communication between both roles depended on it [12]. We were able to identify a series of “sources of parent trust” in the doctor, which would be the evidence cited above. Thus, parents’ trust in doctors is based on the former’s observation that the latter (a) appear to be human, sensitive and empathetic; (b) show transparency and communicative openness; (c) are supportive of parental proactivity; (d) are available for the families at all times.

In our new approach to the data aimed at theorizing on role construction, we have presented trust as a source of emotional strength. Trust undoubtedly acts as a driver for “positive thinking”. According to Ziethut and Bartels, “positive thinking” is one of the most frequently stated ways to cope with caregiving [29]. In their study, this category included citations focused on being optimistic, cherishing each day, and finding the good in the situation. Not only do parents develop trust in the doctors, but also in their own strength, in the fact that they will always have enough energy to cope with the daily situation, in the belief that their children are receiving the right treatment, in the sufficiency of the resources available, or in the effectiveness of the support received from other parents in similar circumstances. Any increase in confidence translates into an increase in what we have called “emotional strength” in our framework.

With the faith category (#6), we have proposed a spiritual dimension of the role experience. In their meta-analysis, Pelentsov et al. found that scientific papers barely cite spiritual and psychological needs compared to the other domains within the supportive care needs framework [9]. The spiritual needs of parents of children with rare diseases push them to search for meaning in their experiences, changing their outlook on what they consider important. For some parents, say the authors, not feeling connected or not ‘belonging’ to a church community because of their child’s disease resulted in them having a crisis of their faith (p. 487).

However, some authors have also pointed to this dimension as a fundamental component of the caregiver’s experience. Noonan and Tennstedt identified three dimensions in the meaning of caregiver: “reordering priorities”, “relationship fidelity” (a sense of feeling useful and needed), and “transcendent beliefs” (a connection to values or religious beliefs) [17]. Each of these correlated significantly—although it was a low/moderate correlation—with indicators of caregiver well-being, related to self-esteem and life satisfaction, although no relationship was detected with mastery or feeling able to handle the situation, nor with “role captivity” or feelings of “loss of self”.

In Noonan and Tennstedt’s study, meaning in caregiving, or, more precisely, the caregivers’ sensation of having found meaning in what they do, was related positively to their own search for meaning [17]. In other words, the more they searched for a transcendent meaning to their activity as caregivers, the more they found it. The more they found it, the greater well-being they reported in their caregiving role. However, most studies on caregiving have not explored the cognitive and emotional aspects of transcendent meaning in the caregiving experience, as if the subject’s “spiritual” aura made researchers reluctant to explore it.

The psychic substratum that we have proposed (existential unease and emotional strength) provides the framework for the construction of the role identity when taking care of a child with a rare disease. This construction, in turn, is governed by the success or failure dynamic in overcoming fear through love. Fear, as we have seen, is the psychological state that underlies “existential unease”, while love is the psychological state that underlies “emotional strength”. In the study by Strehle and Middlemiss, parents with children affected by 4q-syndrome were asked how their child had contributed most to their lives [30]. A majority of 86% (24/28) replied that caring for and bringing up their child had enriched their lives and given them new perspectives, despite all the difficulties encountered and the years of hard work:

The phrases used were: “he is our gift”, “she taught me patience”, “the best thing that ever happened to me”, “she adds pure joy to my life”, “the most loving individual I have ever met”, “she is the sunshine of our life”, “he made me realise I can cope with anything” and “he is a constant reminder of what is important in life”. (p. 196)

These expressions clearly illustrate what it means to experience the parent/caregiver role from the “love” zone.

Regarding role-related behaviours, our results show how parents/caregivers have to display behaviours from the very beginning, regardless of their emotional strength, and when they are already experiencing existential unease. This is how they begin to play their role, developing their caregiver identity with specific behaviours that we have identified in categories #7, #8, #9 and #10. In this sense, our findings concur, to a large extent, with those of Sullivan-Bolyai et al., when they describe the daily tasks associated with the parent/caregiver role, in which they include private proactivity in preference to public proactivity [8].

Even so, the commitment required to care for a child with a rare disease is far greater than the commitment required or the burden placed on a caregiver in a typical parenting situation. Thus, the parent of a child with a rare disease is perceived not only as a parent but also as a caregiver. The parental role is shifted and, as we have seen in the “Results” section, it can be shifted in three directions.

On one hand, the parental role can be extended, adding a caregiving role to a parenthood situation that still prevails. This extension of the parental role is discussed by Broome et al., for example. In their study, which also used grounded theory methodology, they describe how diagnosis challenges parental competency, as they now have to take on a role that requires expanding parental competency, while at the same time developing new skills to manage the newly identified disease [31].

Gillespie et al., using the same grounded theory methodology, seem to implicitly defend the “parental role extension” model, as well as stressing the importance of “fear” as a feature of the parental experience [32]. The last of the five major themes that emerge from their analysis of parents/caregivers of children with food-induced anaphylaxis points in that direction:

- Living with fear: fear due to risk to life; fear following diagnosis; fear as the child’s world expands; fear in the present; and fear for the future.
- Worrying about well-being: risk to development and general well-being; concern for physical, emotional, and social development.
- Looking for control: major strategy for coping with risk; environmental control; control and other people; preparing the child for control.
- Relying on resources: resources used for coping with risk; mothers’ personal resources; support gained from other people; information resources.
- It is hard but it is not: adaptation to the disease’s risks; rare disease integrated into their daily lives.

In those cases where “it is hard but it is not”, where there has been a true adaptation to the health risks or where the disease has been integrated into their daily lives, the process of extending the parental role has been relatively successful.

The second form of role shift takes the form of a separation between the parental role and the caregiver role, with a clear dissociation between the two. Research by Baumbusch et al. illustrates the common challenges experienced by parents of children with rare diseases as they navigate the healthcare system [33]. As the authors say, many rare diseases are diagnosed during childhood, and therefore parents become primary caregivers in addition to their parental role. Their results showed that parents’ role as “expert caregiver” was rarely acknowledged by healthcare providers, pointing to the need to foster more egalitarian relationships. In addition, parents were burdened with the additional role of care coordinator, a role that could be filled formally by a healthcare provider. Lastly, peer support was a key resource in terms of information and emotional support for parents who often begin their journey feeling isolated and alone (p. 80).

However, we have also analysed the third path, and that is the over-dimensioning of the caregiving role. When a rare disease appears in a child, especially if it is chronic, it invariably causes a transition from the parental role to the parental/caregiver role. Both the transition itself and the path subsequently followed by the role should be viewed as dynamic processes. Very appropriately, Poyner-Reed suggests using the construct “career” to refer to it [34]. Thus, the caregiving task would not be composed of a fixed set of experiences, but of a series of changing configurations, with different sources of stress and different coping strategies appearing in each phase. The career construct highlights the different phases of care, paying particular attention to what is shared by the stress processes, and the inherent dynamics at any given time. Caregivers undergo continual changes and restructuring processes. The career construct is particularly suited to capturing this context of change, as it includes both the dynamic movement of caregivers from one status to another and the patterns of change, they experience at each phase.

Lastly, we would like to mention what we consider could be some of the limitations of our study. The sample of parents interviewed may correspond to a specific parenting profile. The parents who have given us their time (remember that the interviews lasted between 60 and 90 min, a very valuable time for people who are heavily burdened by their role as caregivers) are people who, most probably, are living their experience from a place of emotional strength rather than existential unease. According to our findings, parents with experiences based on love (understood as acceptance of their reality) and emotional strength may be more willing to take part in studies that give more visibility to their children’s diseases (acting proactively from their role identity). A future line of research would be to be able to reach parents who cannot go much beyond the existential unease which their children’s disease elicits in them.

## 5. Conclusions

A parent/caregiver role exists only insofar as there is a role identity, that is, insofar as someone defines, identifies or recognizes themselves through the performance of the role. According to our theory of how parents build a role identity around caring for a child with a rare disease, this role identity is the outcome of the—relative and self-perceived—success or failure in progressively overcoming fear through love. Due to its duration and transformative impact, we like to describe this process as navigation between love and fear.

We can basically talk of two types of fear—fear of losing something we think we have or fear of not getting something we want to have. In the context of our study, fear should be understood as a broad-spectrum psychological state that has become the active principle that triggers and sustains all the different manifestations of “existential unease” in which parents find themselves in the first phases of their children’s disease, before and immediately after diagnosis.

Fear may overpower, but love empowers. Ultimately, love is the energy source that not only gives emotional strength but enables this strength to become the foundation for altruistic behaviour. Trust and faith, as defined here, are merely signs that help the parent/caregiver discover and give free rein to love, which is nothing other than the natural acceptance of the reality in which they must live. The role can be played in many different ways, but without a doubt, the healthiest way, both for the parents and for their children, is that in which love has prevailed over fear.

## Data Availability

Data supporting reported results can not be found. Participants exclude access to their data in their consent statement.
